# Effect of Harvesting Ages on Yield and Yield Components of Sugar Cane Varieties Cultivated at Finchaa Sugar Factory, Oromia, Ethiopia

**DOI:** 10.1155/2021/2702095

**Published:** 2021-09-18

**Authors:** Gemechis Dugasa Urgesa, Ebisa Olika Keyata

**Affiliations:** ^1^Department of Chemical Engineering, Wollega University, P.O. Box 38, Shambu, Ethiopia; ^2^Department of Food Science and Nutrition, Wollega University, P.O. Box 38, Shambu, Ethiopia

## Abstract

This study was initiated with the objective of determining the effect of different harvesting ages (12, 13, 14, 15, and 16 months) on yield and yield components of selected the two sugar cane varieties (B52/298 and NCo 334) grown in Finchaa sugar factory, Oromia, Ethiopia. A field experiment was conducted at Finchaa sugarcane plantation using a randomized complete block design of a factorial arrangement of 2 × 5 with three replications. The data were performed using SAS version 9.3, and a significant difference was considered at *p* ≤ 0.05. The results showed that B52/298 variety had a higher estimated recoverable sucrose than NCo 334 variety. The results also indicated that as harvesting ages increase yield, yield components of sugar cane quality are increased. The maximum sugar yield of 1.89-ton ha-1 month-1 was obtained at the harvesting age of 15 months. There was a significant difference (*p* < 0.05) between harvesting age and sugarcane varieties on cane yield, sugar yield, brix percent juice, pol percent, and recoverable sugar. Generally, the findings imply that as harvesting ages in month increase, brix percent juice, estimated recoverable sucrose, and sucrose percentage in both varieties were simultaneously increased. The findings suggested that B52/298 sugar cane variety with harvesting age between 14 and 16 is highly recommended to obtain optimum sugar cane yield and yield components at the tropical areas of Finchaa sugar factory.

## 1. Introduction

Sugarcane (*Saccharum* spp. *hybrids*) is a major industrial source of raw materials for sugar and ethanol production that is cultivated in tropical and subtropical areas around the world [1]. Because of its economic impact on renewable energy production, global interest in sugarcane has increased significantly [2]. Sugarcane is a sun-loving plant that can be grown up to 1600 meters above sea level near the equator and up to 600 meters above sea level between 35° N and S latitudes under a variety of soil and climatic conditions.

Cane quality is one of the most important aspects of sugarcane postharvest management, as it deteriorates in the field due to factors such as ambient temperature, humidity, cane variety, storage time of soluble invertases in cane, and maturity status [3–5]. Age of harvest is one of the most significant factors affecting sugarcane production [6]. Improper harvest age is a chronic issue of preharvest cultural practices, which has a negative impact on cane quality and yield. In addition to this, environmental conditions, management practices, and pest pressure also affect the optimum harvest age of sugarcane and their qualities components [7]. Authors also showed that temperature, solar radiation, relative humidity, and total rainfall are climate variables that account for a significant difference in harvest age among sugarcane-growing countries.

Harvesting cane either underaged or overaged at the improper time results in a loss of cane production, sugar recovery, and low juice consistency [8]. The peak sucrose content of sugarcane at harvest time is affected by different growing environment and plant physiological conditions during the maturation period [9]. Furthermore, the variation among soil on cane fields causes considerable differences in soil moisture holding capacity, degree of drying, and, consequently, the rate at which cane fields ripen [10]. Thus, sugarcane must be harvested at the right time, i.e., at peak maturity, in order to achieve the maximum weight of millable canes achieved with the least amount of field losses possible in the given growing area [11].

Sugar plantations in Ethiopia have a wide range of harvest seasons for all sugar cane varieties, ranging from 18 to 24 months. Some sugar cane cultivars are harvested during early maturity which may significantly affect sucrose content [12]. Information on effects of harvesting ages on yield and yield components of sugarcane varieties cultivated at Finchaa sugar factory Oromia, Ethiopia, is limited. Thus, in this work are reported effects of harvesting ages and sugarcane varieties on cane and sugar yields, brix percent juice, estimated recoverable sucrose, and sucrose/pol percentage in the Finchaa sugar factory.

## 2. Materials and Methods

### 2.1. Description of the Study Area

**Figure 1:**
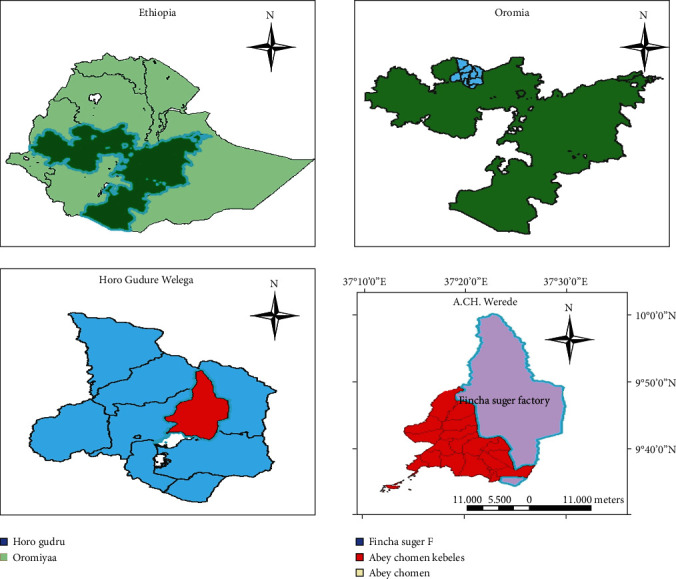
Map of the study area.

The field experiment was conducted at Finchaa sugar estate (Figure 1), which is located in Oromia Regional State, Horro Guduru Wollega Zone, and Abbay Chommen district. The plantation is situated at 9° 30′ to 10° North and 37° 15′ to 37° 30′ East. It is located at about 340 km North West of Addis Ababa at an altitude of about 1350-1650 m above sea. The average annual precipitation at the area is 1309 mm, and the average maximum and minimum daily temperatures are 30.6° and 14.5°C, respectively.

### 2.2. Experimental Design

Factorial combination of two sugarcane varieties (B52/298 and NCo 334) and five harvesting ages (12, 13, 14, 15, and 16 months) were investigated in completely randomized block design (RCBD) with three replications. Each experimental plot was composed of five rows of 9 m length with an area of 65 m^2^ (9 m × 1.45 m × 5 m). All the necessary parameters were collected at an appropriate time throughout the experimental season.

### 2.3. Sample Collection and Preparation

Planting materials represented three budded setts prepared from 8-month age healthy plant crop. The cane setts were made from top one-third to one-half of cane stalks of the varieties at Finchaa sugar factory. These were treated with Ethiozeb (Mancozeb) 80% WP 1 g/l water for 5 minutes to avoid the effects of disease and insect pests. The knife cutters were dipped in ethanol for disinfection at the time of making setts from the cane stalks. The treated setts were planted on the next day to avoid moisture loss before planting. All the agronomic practices were kept uniform in all treatments. Other recommended cultural practices including fertilization (52.5 kg DAP/diammonium phosphate and UREA 87.5 kg for 0.35 ha of experimental area used) and irrigation application, earthing up, and weeding (using postemergency herbicide) were done.

### 2.4. Methods of Data Collections

At various ages, 30 stalks from each harvestable plot were randomly selected and measured for cane yields, sugar yields, Brix percent juice, estimated recoverable sucrose, and sucrose/pol % at Finchaa sugar factory farm.

#### 2.4.1. Cane Yields

Cane yield (t/ha) was estimated from the middle two rows and was calculated on a hectare basis by multiplying number of millable cane stalk and average stalk weight.

(1) $$\begin{array}{c} \text{Cane yield }\left(\text{ton}/\text{ha}\right)=\frac{\text{number of millable canes}}{\text{ha}}*\text{stalk weight }\left(\text{kg}\right). \end{array}$$

#### 2.4.2. Sugar Yields

Estimated sugar yield (ESY) tone per hectare was calculated as follows:

(2) $$\begin{array}{c} \text{SY }\left(\text{t}/\text{ha}\right)=\text{CYH }\left(\text{t}/\text{ha}\right)\times \text{ERS }\left(\%\right), \end{array}$$

where SY is the sugar yield, CYH is the cane yield per hectare, and ERS % is the estimated recoverable sugar percent.

#### 2.4.3. Brix Percent Juice

Brix % juice for all samples of treatment combinations was determined by using Brix hydrometer standard methods. The juice solution was transferred into cylinder then, and degree Brix and temperature were determined. The Brix of extracted juice was corrected from temperature correction table.

The reading by refractor meter Brix reading recorded = corrected Brix.

The reading by hydrometer Brix % was corrected to Brix % juice as follows:

(3) $$\begin{array}{c} \text{Brix}\%\text{juice }\left(B\right)=b\;\left(6–0.0125F\right), \end{array}$$

where *B* is the Brix % juice, *b* is the corrected Brix, and *F* is the fiber % cane.

#### 2.4.4. Estimated Recoverable Sucrose

Sugar recovery is the amount of sugar recovered from a fixed amount of sugarcane during the crushing process, and it was calculated as described by Gamechis and Vighneswara [13].

(4) $$\begin{array}{c} \text{ERS}\%=\left\{\text{pol}\%\text{juice}-\left(\text{Brix}\%\text{juice}-\text{pol}\%\text{juice}\right)\text{NSF}\right\}*\text{CF}, \end{array}$$

where NSF is nonsugar factor with constant value of 0.70 and CF is cane factor with constant value of 0.57.

#### 2.4.5. Sucrose/Pol Percentage

Pol percent juice was determined by using Polari meter as described by Anonymous [14]. Pol percent was calculated from Schmitz's table using Home's Dry lead methods 1961 according to the standard procedure.

(5) $$\begin{array}{c} \text{Pol}\%\text{juice}=p\;\left(6–0.0125F\right),\\ \text{Dry substance}\%\text{cane}=100\text{ g}–\text{weight loss in g}, \end{array}$$

where *D* is dry substance % cane, *b* is the corrected Brix, *P* is the corrected pol, and *F* is the fiber % cane.

### 2.5. Statistical Analysis

The triplicate data were subjected to analysis of variance (ANOVA). All the statistical analyses were performed using SAS version 9.3, and significance difference was considered at *p* ≤ 0.05. Fisher's least significant difference (LSD) was used for mean comparison tests to identify significant differences among means (*p* ≤ 0.05).

## 3. Results and Discussion

### 3.1. Effect of Sugar Cane Varieties on Yield and Yield Components

The results on the cane yield, sugar yield, brix, estimated recoverable sucrose, and sucrose percentage of sugar cane varieties (B52/298 and NCo 334) are presented in Table 1. There had a non significant (*p* > 0.05) difference in the cane yield, sugar yield, and sucrose percentage contents between the two sugar cane varieties. However, there was a significant variation (*p* < 0.05) in the brix and sugar recovery between the two varieties. The variations in brix and estimated recoverable sucrose between B52/298 and NCo 334 varieties of sugarcane might be due to genetic, morphological, and physiological factors (Shahzad et al., [15]). In addition to this, the ability to synthesize and store soluble solid compounds may differ depending on the brix of sugar cane varieties. This finding also highlighted that B52/298 variety had higher estimated recoverable sucrose than NCo 334 variety.

### 3.2. Effect of Sugar Cane Harvesting Ages on Yield and Yield Components

The effect of harvesting ages of sugar cane in months on cane yield, sugar yield, Brix, estimated recoverable sucrose, and sucrose are indicated in Table 2.

There was a nonsignificant (*p* > 0.05) difference in the cane yields among harvesting ages in 12, 13, and 14 months and also between 15 and 16 months. However, there was significant (*p* < 0.05) differences in the cane yields when compared with harvesting ages 15 and 16 with 12, 13, and 14 months. The findings also showed that as harvesting ages in month increase, cane yield simultaneously increased. Similar results were reported by Hagos et al. [7] who found that increasing the harvest age from 10 to 14 months resulted in a considerable increase in cane output.

Harvesting age in months had significantly (*p* < 0.05) influenced on sugar yield. The result showed that as harvesting age from 12 to 15 months increases, sugar yield also increases. However, sugar yield begins to diminish after 16 months. This could be owing to a drop in cane output due to the lodging effect, which has a direct impact on sugar yield, even while quality criteria continue to improve with age. Beside this, sugar yields depend on cane yield with its component stalk population, length, and diameter [16].

There was no significant (*p* > 0.05) differences in the brix and estimated recoverable sucrose between harvested ages in 12 and 13 months. However, harvesting ages significantly (*p* < 0.05) influence on brix and estimated recoverable sucrose among age in 14, 15, and 16 months. The findings showed that brix juices and estimated recoverable sucrose percentage increase when sugar cane harvesting ages increased. The increase in brix with harvesting age is related to the continual accumulation of solids as harvest age progresses until the end of the harvesting season [17]. The findings of this study are consistent with the result of Cardozo and Sentelhas [10], who reported that harvesting age had a substantial impact on Brix percent juice. These results are also in agreement with Shikanda et al. [18] who reported that harvest age showed highly significant influence on Brix, sucrose, and purity percentage.

Harvesting sugar cane ages exhibited a significant (*p* < 0.05) effect on sucrose percentage. The sucrose content of the sugar cane increased from 12.03 to 13.72% with an increase harvesting ages in months from 12 to 16. The findings strongly suggested that sugarcane harvesting at the right time is required to achieve maximum sucrose accumulation and sugar output in the tropical area of Finchaa sugar factor with the least feasible field losses under the growing conditions.

### 3.3. Interaction Effects between Sugar Cane Varieties and Harvesting Ages on Cane Yields and Yield Components

#### 3.3.1. Cane Yield

Sugar cane varieties and harvesting age had a significant (*p* < 0.05) impact on cane yield ton ha-1 month-1 (Table 3). The maximum value of cane yield ton ha-1 month-1 was recorded at the harvesting age of 16 months for both sugarcane varieties. However, the minimum value was found at the harvesting age of 13 months for both varieties.

#### 3.3.2. Sugar Yield

Sugar yield was significantly (*p* < 0.05) affected by interaction of variety and harvesting ages (Table 4). However, there was no significant (*p* > 0.05) difference at the harvesting age of 12 and 16 months between both varieties (B52/298 and NCo 334). The maximum sugar yield ton ha-1 month-1 (1.89) was observed from the variety B52/298 at the harvesting age of 15 months. This might be due to the contribution of maximum cane yield at the age of 15 months and the increasing effect of quality parameters with increasing age. Hagos et al. [7] also confirmed that the highest cane yield and sugar yield are obtained because of the increasing effect of longer harvest ages on yield components and quality parameters. However, sugar yield begins to diminish after 16 months. This could be owing to a drop in cane output due to the lodging effect, which has a direct impact on sugar yield, even while quality criteria continue to improve with age.

#### 3.3.3. Brix Percent Juice

The harvest age and sugarcane cultivars had a highly significant (*p* < 0.01) effect on brix percent juice (Table 5). The findings showed that at the age of 16 months, the variety NCo 334 had the highest brix % (19.52) while low was recorded at harvesting 12 in the B52/298 variety. The Ethiopian's brix percent juice in both sugar cane varieties (B52/298 and NCo 334) from Finchaa sugar factory was similar to that reported for sugarcane cultivars produced in Egypt (2.8 mg/g) [19].

#### 3.3.4. Estimated Recoverable Sucrose (ERS)

There was a significant (*p* < 0.05) interaction between harvesting age and varieties on estimated recoverable sucrose (ERS) (Table 6). In this study, the variety B52/298 had the highest ERS % (12.78) when harvested at 16 months. The increase in sugar recovery percentage is mainly due to the increase in sucrose percentage. The findings showed that when the age sugarcane varieties increase, recoverable sucrose increases in both varieties. A similar result was also reported by Hagos et al. [7].

#### 3.3.5. Sucrose Percentage

On percentage of sucrose, there was a significant (*p* > 0.05) difference between harvesting ages and varieties (Table 7). The results showed that sugarcane collected between the ages of 15 and 16 months resulted in a large and ascending increase in sucrose % in both the B52/298 and NCo 334 cultivars. This might be due to accumulation of more sucrose as the harvest age approaches. The results were consistent with three sugarcane varieties produced in Egypt that were harvested at 10, 11, 12, 13, and 14 months of age [17].

## 4. Conclusions

In these study effects of varieties, harvesting ages and their interaction on yields and yield components of sugarcane cultivated at Finchaa sugar factory, Oromia, Ethiopia, were investigated. The notable findings showed that B52/298 sugar cane variety exhibits good amount of cane yield, sugar yield, estimated recoverable sucrose, and sucrose percentage. The results also indicated that as harvesting ages increased yield, yield components of sugar cane quality are increased. The findings also showed that as harvesting ages in month increase, Brix percent juice, estimated recoverable sucrose, and sucrose percentage in both varieties were simultaneously increased. The findings suggested that in terms of yield components, B52/298 sugar cane varieties with harvesting age between 14 and 16 are highly recommended to obtain optimum sugar cane yield and yield components at the tropical areas of Finchaa sugar factory. Future research should conduct across years with extending harvest age beyond 16 months to ascertain the consistency of the recommendation.

## Figures and Tables

**Table 1:** Effect of sugar cane varieties on yield and yield components.

Varieties	Cane yield (ton/ha/month)	Sugar yield (ton/ha/month)	Brix % juice	Recoverable sucrose (%)	Sucrose %
B52/298	11.34^a^	1.23^a^	15.38^b^	11.31^a^	12.03^a^
NCo 334	10.94^a^	1.20^a^	15.72^a^	11.02^b^	11.92^a^
CV	1.97	2.85	0.11	1.03	0.96
LSD	0.49	0.078	0.039	0.26	0.26

**Table 2:** Effect of sugar cane harvesting ages on yield and yield components.

Age in months	Cane yield (ton/ha/month)	Sugar yield (ton/ha/month)	Brix % juice	Estimated recoverable sucrose (%)	Sucrose %
12	11.34^b^	1.23^e^	15.38^d^	11.31^d^	12.03^d^
13	11.13^b^	1.35^d^	15.87^d^	11.47^cd^	12.32^c^
14	11.39^b^	1.49^c^	16.83^c^	11.59^c^	12.63^b^
15	12.08^a^	1.89^a^	17.68^b^	11.98^b^	13.54^a^
16	12.16^a^	1.67^b^	19.03^a^	12.78^a^	13.72^a^
CV	1.41	3.42	2.32	0.82	0.81
LSD	0.31	0.10	0.74	0.18	0.20

**Table 3:** Interaction effect of varieties and harvesting ages on cane yields.

Cane yield					
Varieties	Harvesting ages (months)				
	12	13	14	15	16
B52/298	11.34^cde^	11.13^fe^	11.39^cd^	12.08^a^	12.16^a^
NCo 334	10.94^fg^	10.86^g^	11.24^de^	11.52^cb^	11.64^b^
LSD	0.25				
CV	1.27				

LSD: least significant difference; CV: coefficient of variation. Means within a line followed by the same letter (s) are not significantly different.

**Table 4:** Interaction effect of varieties and harvesting ages on sugar yields.

Sugar yield					
Varieties	Harvesting ages				
	12	13	14	15	16
B52/298	1.23^f^	1.35^e^	1.49^d^	1.89^a^	1.67^c^
NCo 334	1.20^f^	1.24^f^	1.32^e^	1.78^b^	1.62^c^
LSD	0.07				
CV	2.64				

SE: standard error; CV: coefficient of variation. Means within a column followed by the same letter (s) are not significantly different.

**Table 5:** Interaction effect of varieties and harvesting ages on Brix percent juice.

Brix % juice					
Varieties	Harvesting ages				
	12	13	14	15	16
B52/298	15.38^g^	15.87^f^	16.83^de^	17.68^c^	19.03^b^
NCo 334	15.72^fg^	16.43^e^	16.96^d^	17.94^c^	19.52^a^
LSD	0.47				
CV	1.59				

SE: standard error; CV: coefficient of variation. Means within a line followed by the same letter (s) are not significantly different.

**Table 6:** Interaction effect of varieties and harvesting ages on estimated recoverable sugar (%).

Estimated recoverable sugar (%)					
Varieties	Harvesting ages				
	12	13	14	15	16
B52/298	11.31^fg^	11.47^de^	11.59^cd^	11.98^b^	12.78^a^
NCo 334	11.02^h^	11.24^g^	11.43^ef^	11.63^c^	12.04^b^
LSD	0.14				
CV	0.7				

SE: standard error; CV: coefficient of variation. Means within a line followed by the same letter (s) are not significantly different.

**Table 7:** Interaction effect of varieties and harvesting ages on sucrose (%).

Sucrose %					
Varieties	Harvesting ages				
	12	13	14	15	16
B52/298	12.03^gh^	12.32^f^	12.63^e^	13.54^b^	13.72^a^
NCo 334	11.92^h^	12.14^g^	12.37^f^	12.96^d^	13.24^c^
LSD	0.15				
CV	0.71				

SE: standard error; CV: coefficient of variation. Means within a line followed by the same letter (s) are not significantly different.

## Data Availability

The data used to support the findings of this study are available from the corresponding author upon request.
